# Supplementary material for: Endothelial alpha globin is a nitrite reductase
Source: Nat Commun. 2022 Oct 27;13:6405. doi: 10.1038/s41467-022-34154-3 (PMC9613979; doi:10.1038/s41467-022-34154-3)
Supplement: Supplementary file 1 — Supplementary Information [file 41467_2022_34154_MOESM1_ESM.pdf]

Supplemental Figures for:

Endothelial alpha globin is a nitrite reductase

T.C. Stevenson Keller IV[1,2]
Christophe Lechauve[3]
Alexander S. Keller[1,4]
Gilson Brás Broseghini-Filho[1,5]
Joshua T. Butcher[6]
Henry R. Askew Page[1]
Aditi Islam[1]
Zhe Yin Tan[1]
Leon J. DeLalio[1,4]
Steven Brooks[7]
Poonam Sharma[8]
Kwangseok Hong[9]
Wenhao Xu[10]
Alessandra Simão Padilha[5]
Claire A. Ruddiman[1,4]
Angela K. Best[1]
Edgar Macal[1]
Daniel B. Kim-Shapiro[11]
George Christ[8]
Zhen Yan[1]
Miriam M. Cortese-Krott[12]
Karina Ricart[13]
Rakesh Patel[13]
Timothy P. Bender[14]
Swapnil K. Sonkusare[1,2]
Mitchell J. Weiss[3]
Hans Ackerman[7]
Linda Columbus[2,15]
Brant E. Isakson[1,2] *

[1] Robert M Berne Cardiovascular Research Center, University of Virginia School of Medicine, Charlottesville, VA
[2] Department of Molecular Physiology and Biophysics, University of Virginia School of Medicine, Charlottesville, VA
[3] Department of Hematology, St. Jude Children's Research Hospital, Memphis, TN
[4] Department of Pharmacology, University of Virginia School of Medicine, Charlottesville, VA
[5] Department of Physiological Sciences, Federal University of Espirito Santo, Brazil
[6] Department of Physiological Sciences, College of Veterinary Medicine, Oklahoma State University, Stillwater, OK
[7] Physiology Unit, Laboratory of Malaria and Vector Research, National Institute of Allergy and Infectious Diseases, Bethesda, MD
[8] Department of Biomedical Engineering, University of Virginia, Charlottesville, VA
[9] Department of Physical Education, College of Education, Chung-Ang University, Seoul South Korea
[10] Transgenic Mouse Facility, Department of Medicine, University of Virginia School of Medicine, Charlottesville, VA
[11] Department of Physics, Translational Science Center, Wake Forest University, Winston-Salem, NC
[12] Cardiovascular Research Laboratory, Division of Cardiology, Pneumology and Angiology, Medical Faculty, Heinrich Heine University, Düsseldorf, Germany
[13] Department of Pathology, University of Alabama at Birmingham, Birmingham, AL
[14] Department of Microbiology, Immunology and Cancer, University of Virginia School of Medicine, Charlottesville, VA
[15] Department of Chemistry, University of Virginia, Charlottesville, VA

** To whom correspondence should be addressed*
University of Virginia School of Medicine
Charlottesville, VA 22908 USA
E: brant@virginia.edu
P: 434-924-2093

Supplemental Figure 1:

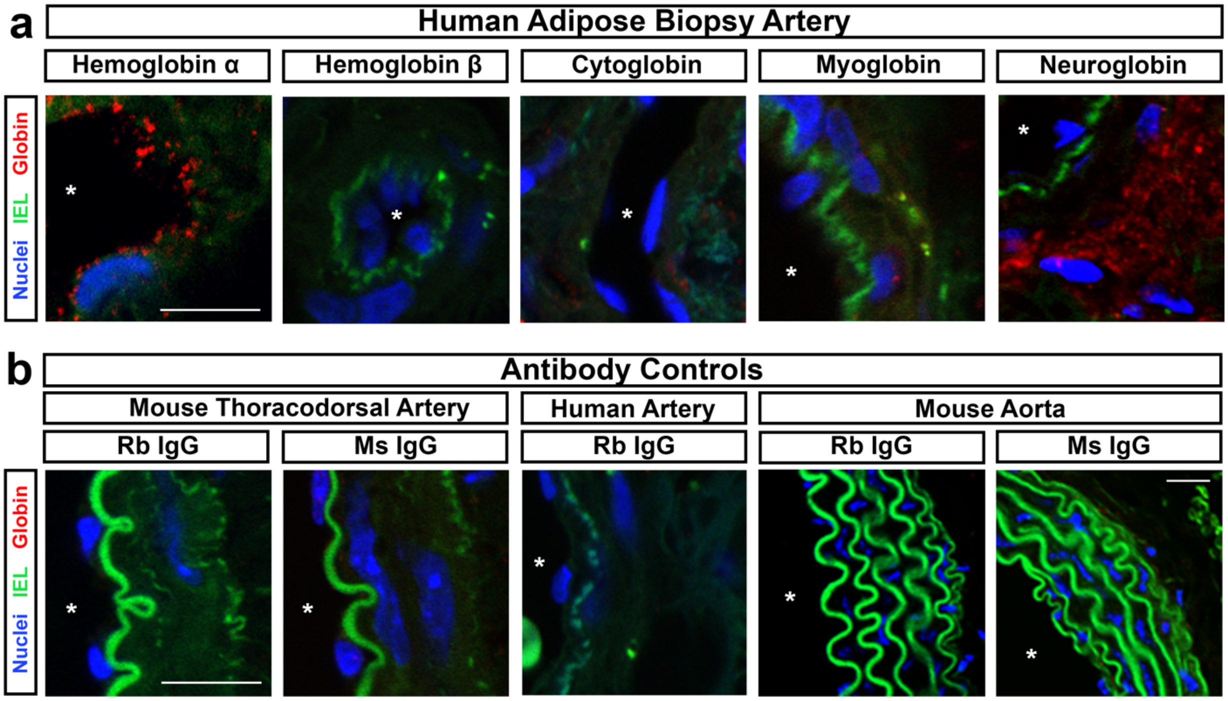

SUPPLEMENTAL FIGURE 1: Alpha globin is expressed in the endothelium of arterioles from human adipose biopsy samples. (**a**) Expression of alpha globin in the endothelium is observed from human adipose biopsy samples. No other globin was detected in the endothelium; neuroglobin signal was observed in the adventitial layers. (**b**) Antibody controls for the positive signals seen in the tissues in Main Text Figure 1 and in this figure, demonstrating the specificity of the staining. In all images, an asterisk indicates the lumen of the vessel. The scale bars in panel **a** represent 10 μm, those in panel **b** represent 25 μm. The images are representative of the analysis of at least four individuals in each case.

Supplemental Figure 2:

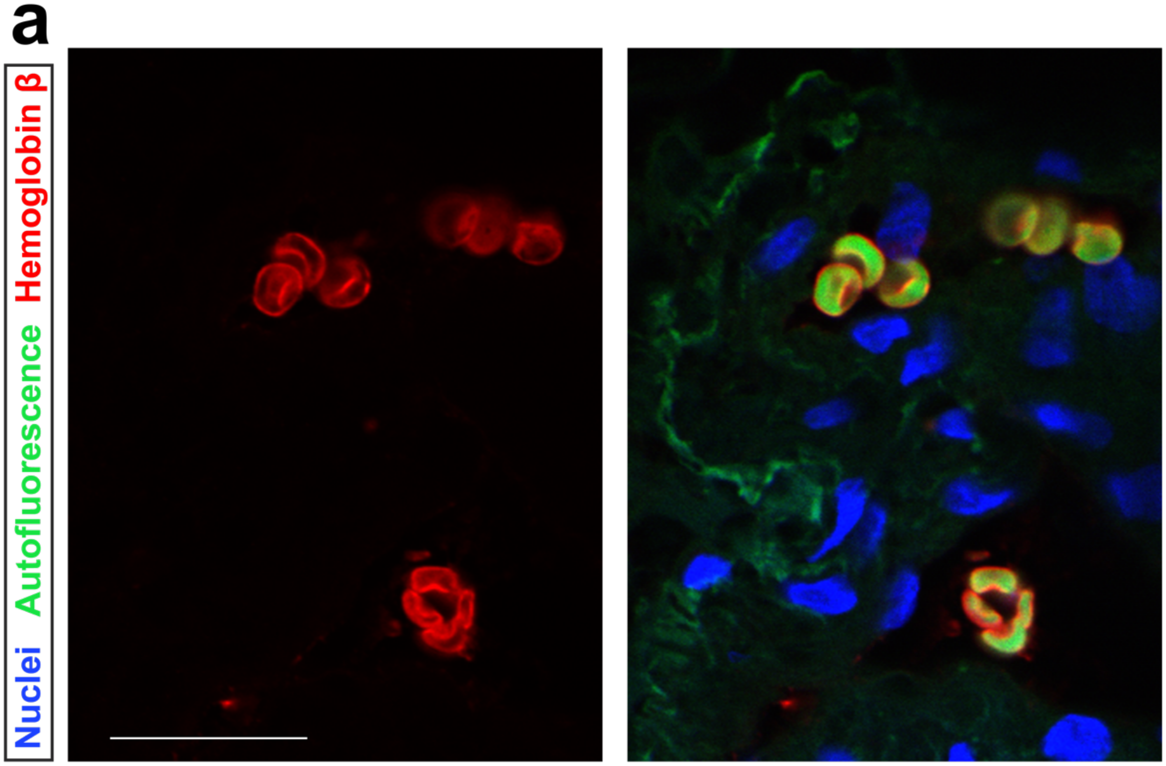

SUPPLEMENTAL FIGURE 2: Staining for hemoglobin β in erythrocytes, as an immunohistochemistry positive control. (**a**) Positive signal (red) for hemoglobin β is observed in erythrocytes captured in a section of a human adipose biopsy. Erythrocytes are strongly autofluorescent (green), and nuclei are stained with DAPI (blue). These results are representative of at least 3 independent experiments. The scale bar represents 30 μm.

Supplemental Figure 3:

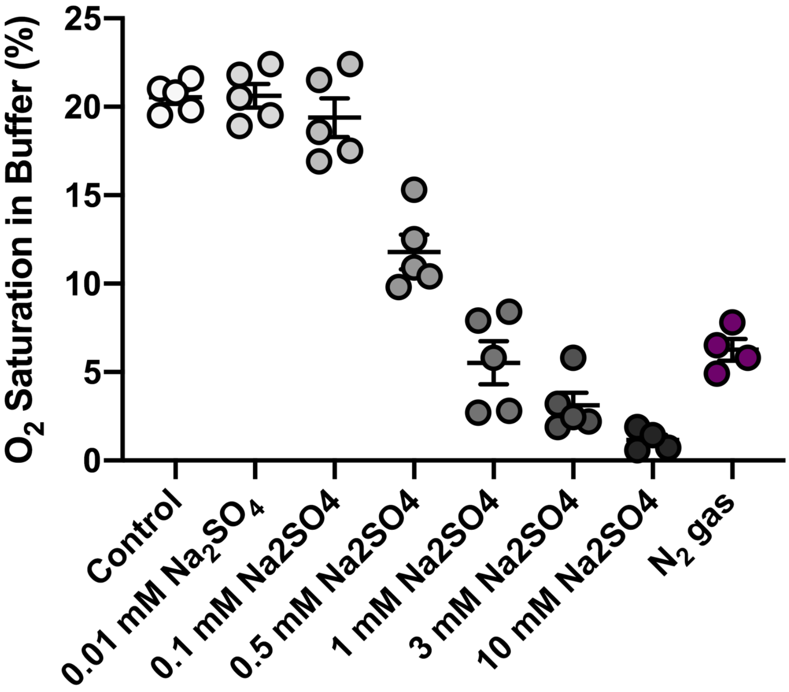

SUPPLEMENTAL FIGURE 3: Oxygen content of buffer treatments with sodium dithionite as used in the experiments. Increasing doses of $Na_2S_2O_4$ decrease the O_2 saturation in the KREBS-based buffer used for the vasoreactivity experiments. The single dose of 1 mM $Na_2S_2O_4$ used in our studies produces an aqueous O_2 saturation similar to that obtained by bubbling the solution with pure nitrogen gas. For each condition, the O_2 saturation was measured in n = 5 samples. Bar graphs are centered on mean with error bars denoting standard error. Source data are provided in the source data file.

Supplemental Figure 4:

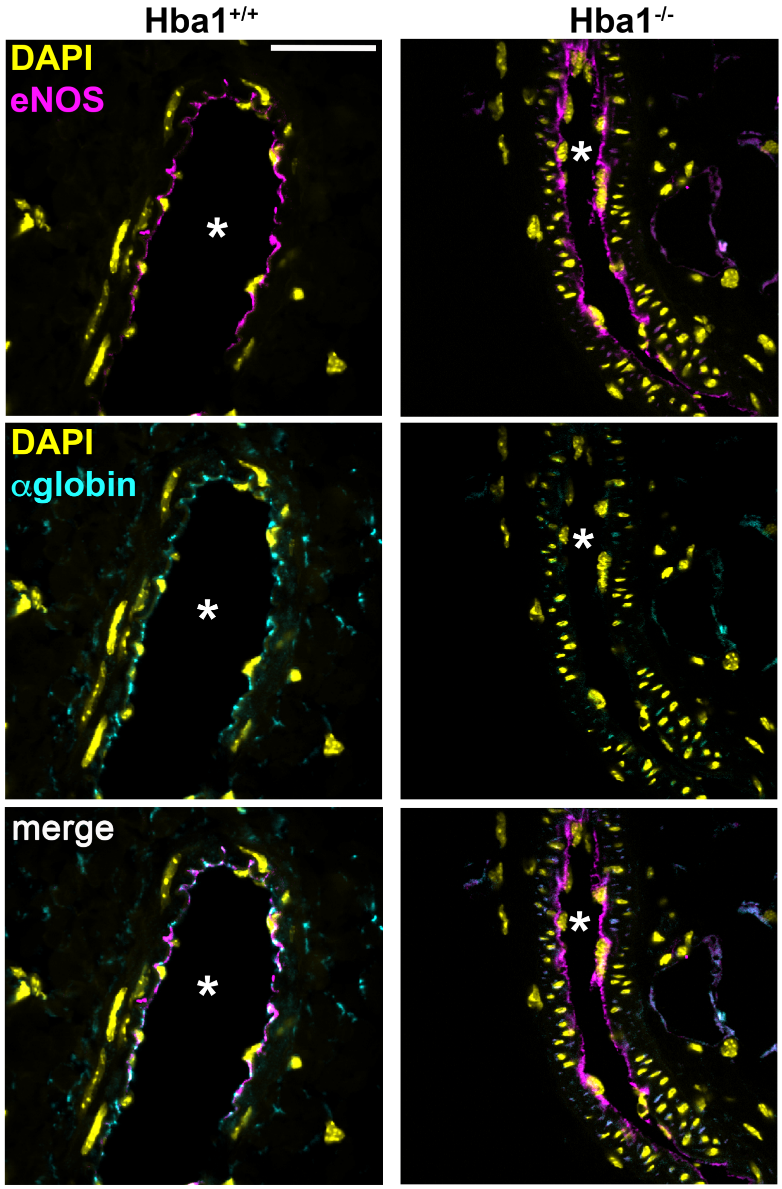

SUPPLEMENTAL FIGURE 4: Loss of endothelial alpha globin in the Hba1$^{-/-}$ *model.* Immunohistochemical staining for eNOS (top, magenta) and alpha globin (middle, cyan) in the *Hba1*$^{+/+}$ (left) and *Hba1*$^{-/-}$ (right) animals. The bottom row shows all channels (including that for DAPI [yellow]) merged. Alpha globin signal is absent from the endothelium of *Hba1*$^{-/-}$ vessels. In each image, an asterisks indicates the lumen of the vessel. The scale bar in the top left image represents 50μm. Images are representative of n = 5 samples analyzed for each genotype.

Supplemental Figure 5:

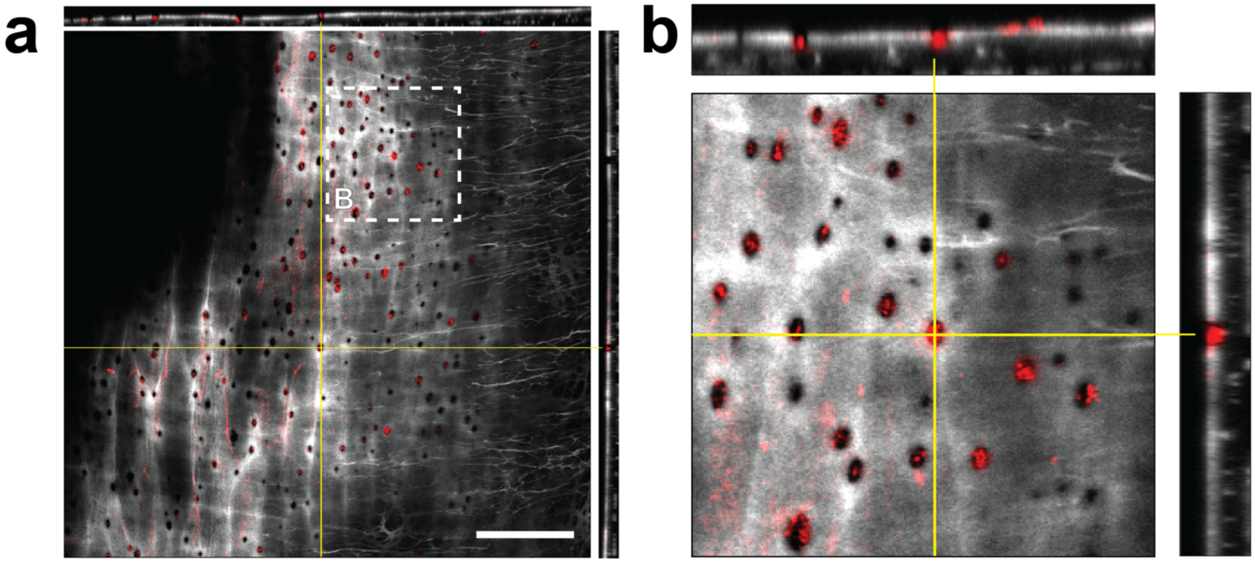

SUPPLEMENTAL FIGURE 5: Localization of alpha globin to the holes in the internal elastic lamina. Confocal microscopic analysis of the expression of alpha globin in *en face* preparations shows that alpha globin is localized to the holes in the internal elastic lamina at a site called the myoendothelial junction. (**a**) Low-power image showing many holes in the internal elastic lamina (gray) with alpha globin (red fluorescent signal) expression inside. (**b**) High-power image of the boxed region in panel **a**. Yellow lines indicate the planes of analysis in the XZ and YZ projections shown, respectively, on the top and right of the main image. Scale bar in **a** represents 50 µm. These images are representative of 6 independent experiments.

Supplemental Figure 6:

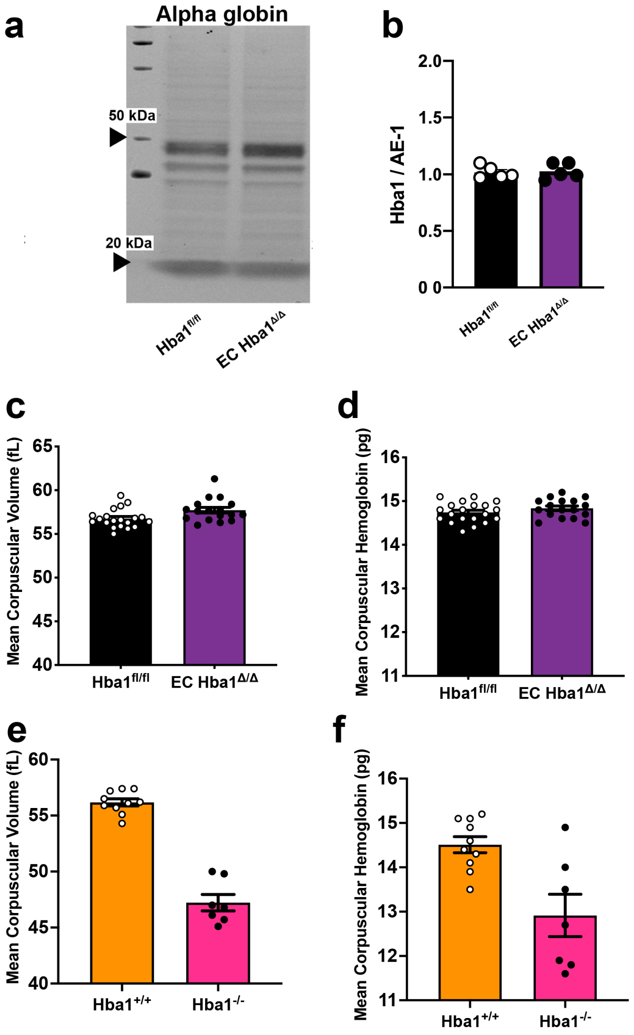

SUPPLEMENTAL FIGURE 6: Additional hematologic measurements for the EC Hba1$^{\Delta/\Delta}$ model. (**a**) Expression of alpha globin within the erythrocytes was unchanged in the EC *Hba1*$^{\Delta/\Delta}$ mice. This uncropped Western blot of alpha globin protein (~18 kDa) shows similar expression levels in the *Hba1*$^{fl/fl}$ and EC *Hba1*$^{\Delta/\Delta}$ mice. (**b**) Normalization of alpha globin protein to AE1 protein levels is shown (n = 5 for each quantification). (**c, d**) Mean corpuscular volume (MCV) (**c**) or mean corpuscular hemoglobin (MCH) (**d**) were not changed as a result of endothelial-specific (*Cdh5-Cre*^{ERT2}) recombination of the *Hba1*$^{fl/fl}$ allele. (**e**, **f**) In the neomycin-insertion global deletion (*Hba1*$^{-/-}$) mice, both MCV (**e**) and MCH (**f**) are decreased compared to WT controls. For these experiments, n = 20 *Hba1*$^{fl/fl}$ mice, n = 17 EC *Hba1*$^{\Delta/\Delta}$ mice, n = 10 *Hba1*$^{+/+}$ mice, and n = 7 *Hba1*$^{-}$

$^{/-}$ mice were used. Bar graphs are centered on mean with error bars denoting standard error. Source data are provided in the source data file.

Supplemental Figure 7:

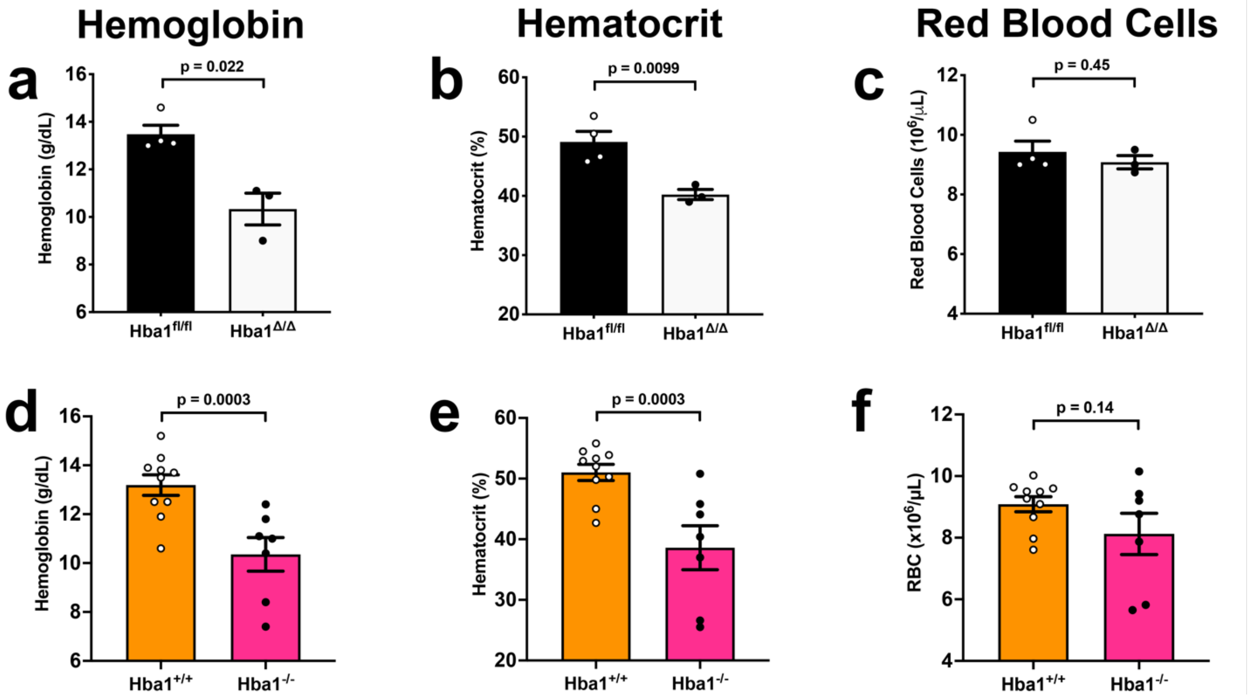

SUPPLEMENTAL FIGURE 7: Blood parameters of mice with global deletion of Hba1 show anemic phenotype. Hemoglobin (**a, d**), hematocrit (**b, e**), and total RBC (**c, f**) show the same patterns in a global *Hba1* KO (*Hba1^fl/fl^; Sox2-Cre*, denoted as $Hba1^{\Delta/\Delta}$) and a model of alpha thalassemia ($Hba1^{-/-}$). For these experiments, n = 4 $Hba1^{fl/fl}$ mice, n = 3 $Hba1^{\Delta/\Delta}$ mice, n = 10 $Hba1^{+/+}$ mice, and n = 7 for $Hba1^{-/-}$ mice were used. Results were compared with one-sided t-tests. Bar graphs are centered on mean with error bars denoting standard error. Source data are provided in the source data file.

Supplemental Figure 8:

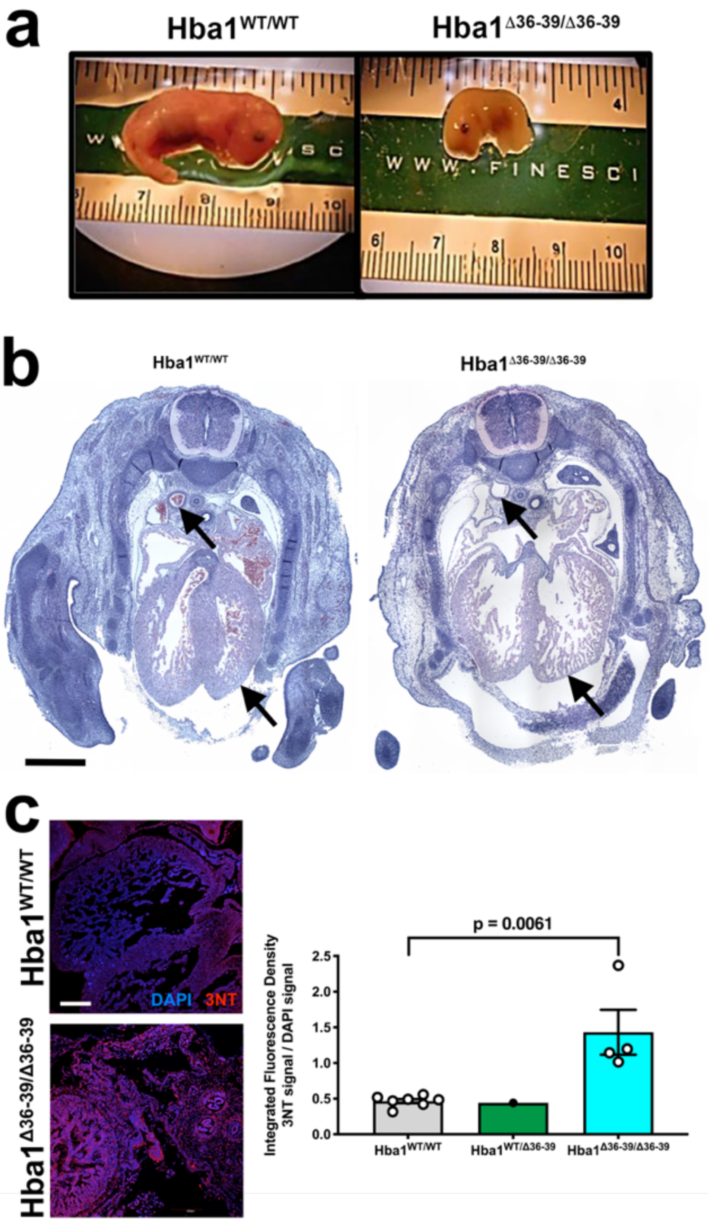

SUPPLEMENTAL FIGURE 8: Homozygous Hba1$^{\Delta 36\text{-}39}$ mutation is embryonically lethal, with pathologic nitrotyrosine formation. (**a**) Embryos with the homozygous Hba1$^{\Delta 36\text{-}39}$ mutation are already deceased at approximately E14.5, showing stunted growth. (**b**) Gross histology shows cardiac non-compaction, lack of blood, and dilated major vessels (arrows). The scale bar represents 5 mm. This image is representative of 4 independent animals. (**c**) Immunostaining for 3-nitrotyrosine (a pathologic marker of excess reactive nitrogen species), shows a large increase

in signal in embryos with homozygous Hba1$^{\Delta 36\text{-}39}$ mutation, as compared to WT or heterozygous embryos. Heart and trunk vasculature is shown. The scale bar represents 20 µm. For the nitrotyrosine quantification in panel **c**, n = 7 *Hba1*$^{WT/WT}$ mice, n = 1 *Hba1*$^{WT/\Delta 36\text{-}39}$ mice, and n = 4 *Hba1*$^{\Delta 36\text{-}39/\Delta 36\text{-}39}$ mice were used. Results for *Hba1*$^{WT/WT}$ and *Hba1*$^{\Delta 36\text{-}39/\Delta 36\text{-}39}$ mice were compared with a one-sided t-test. Bar graphs are centered on mean with error bars denoting standard error. Source data are provided in the source data file.

Supplemental Figure 9:

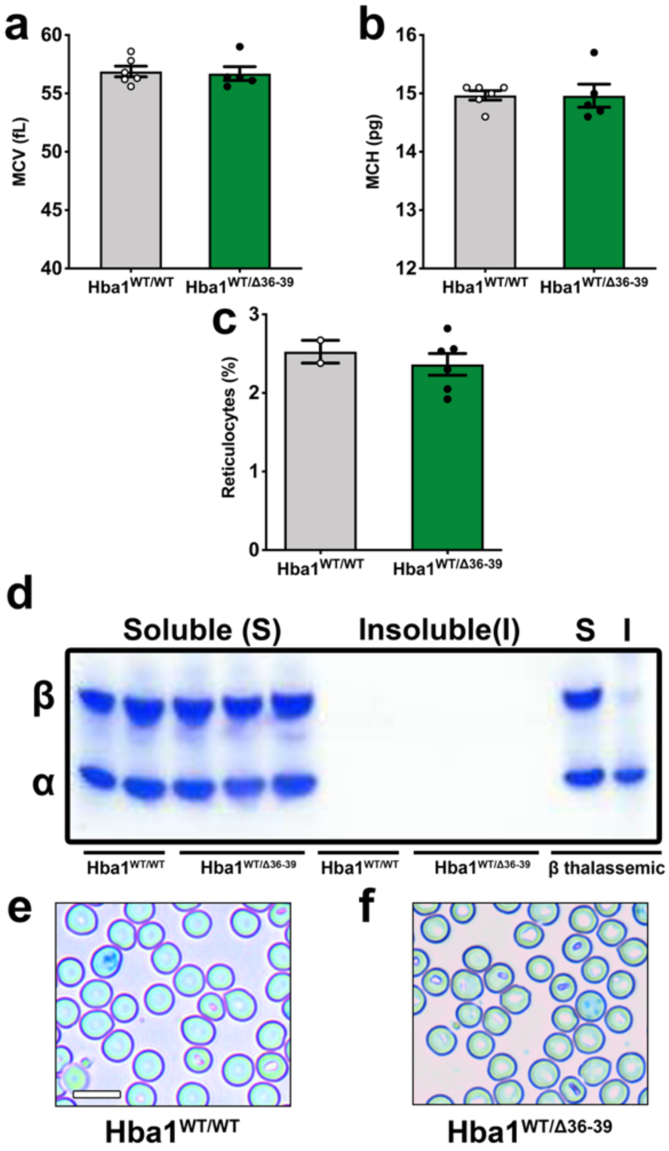

SUPPLEMENTAL FIGURE 9: Hba1$^{WT/\Delta36\text{-}39}$ adults display unchanged red cell and reticulocyte characteristics, with no observed insoluble hemoglobin. (**a–c**) Blood cell parameters, including mean corpuscular volume (**a**), mean corpuscular hemoglobin (**b**), and number of reticulocytes (**c**), did not differ between WT and heterozygous *Hba1*$^{\Delta36\text{-}39}$ mutants. (**d**) Blood samples from WT and *Hba1*$^{\Delta36\text{-}39}$ mutants showed no insoluble hemoglobin. The band corresponding to beta globin is at the top of the gel; the lower band is alpha globin. Insoluble alpha globin was observed in control

sample from a a model of beta thalassemia (far right). This experiment is representative of 3 independent experiments on the same samples. (**e**, **f**) Cresyl blue staining of a blood smear, demonstrating that heterozygous *Hba1*$^{WT/\Delta 36\text{-}39}$ mutants showed no appreciable increase in reticulocytes or precipitated hemoglobin as compared to *Hba1*$^{WT/WT}$ controls. The scale bar represents 5 μm. For the experiments in panels **a** and **b**, n = 6 *Hba1*$^{WT/WT}$ mice, n = 5 *Hba1*$^{WT/\Delta 36\text{-}39}$ mice were used. For the experiments in panel **c**, n = 2 *Hba1*$^{WT/WT}$mice and n = 6 *Hba1*$^{WT/\Delta 36\text{-}39}$ mice were used. Means were compared with a t-test. Bar graphs are centered on mean with error bars denoting standard error. Source data are provided in the source data file.

Supplemental Figure 10:

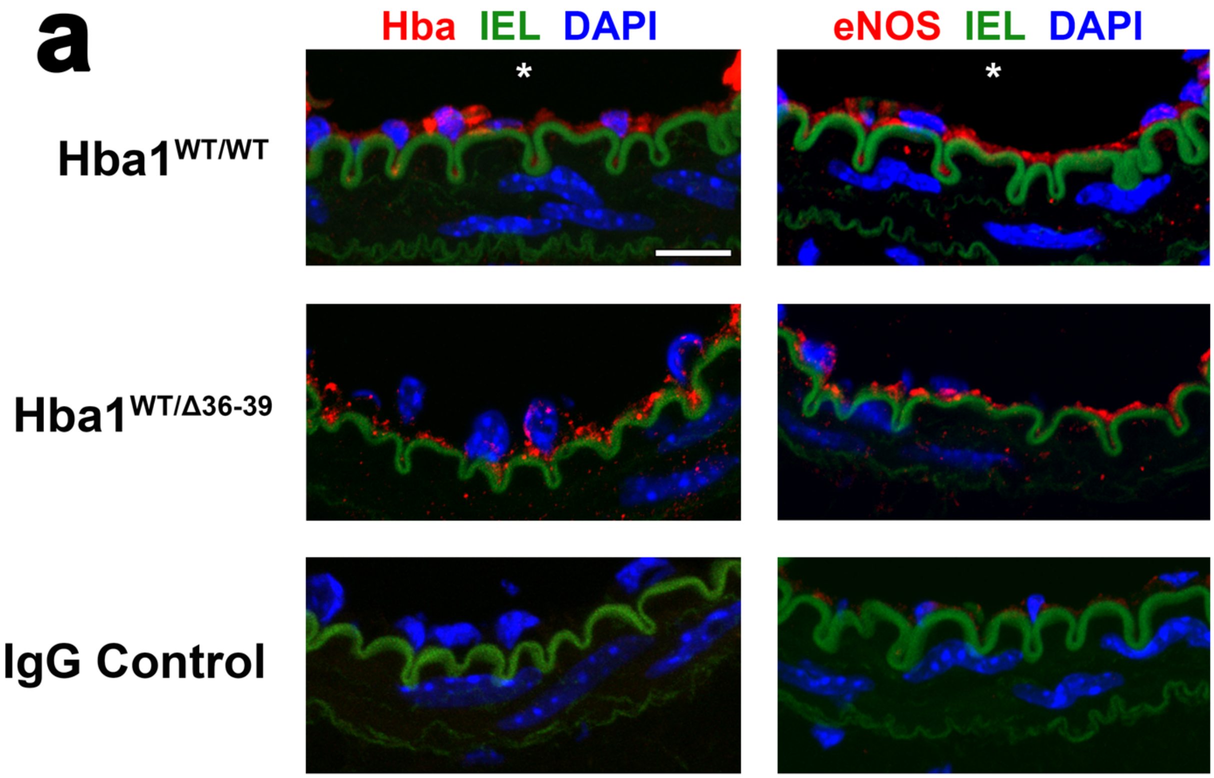

SUPPLEMENTAL FIGURE 10: a, Alpha globin and eNOS are expressed at similar levels in Hba1$^{WT/WT}$ and Hba1$^{WT/\Delta36\text{-}39}$ adult tissues. (a) Immunostaining controls for PLA. Alpha globin (left) and eNOS (right) are expressed and detectable at similar levels in the thoracodorsal arteries of *Hba1*$^{WT/WT}$ and *Hba1*$^{WT/\Delta36\text{-}39}$ mice. The scale bar represents 20 μm. The sections in the bottom row are stained with non-reactive IgG from the primary antibody host animal and shows no signal for either antigen. These images are representative of four individual animals from this experiment.

Supplemental Figure 11:

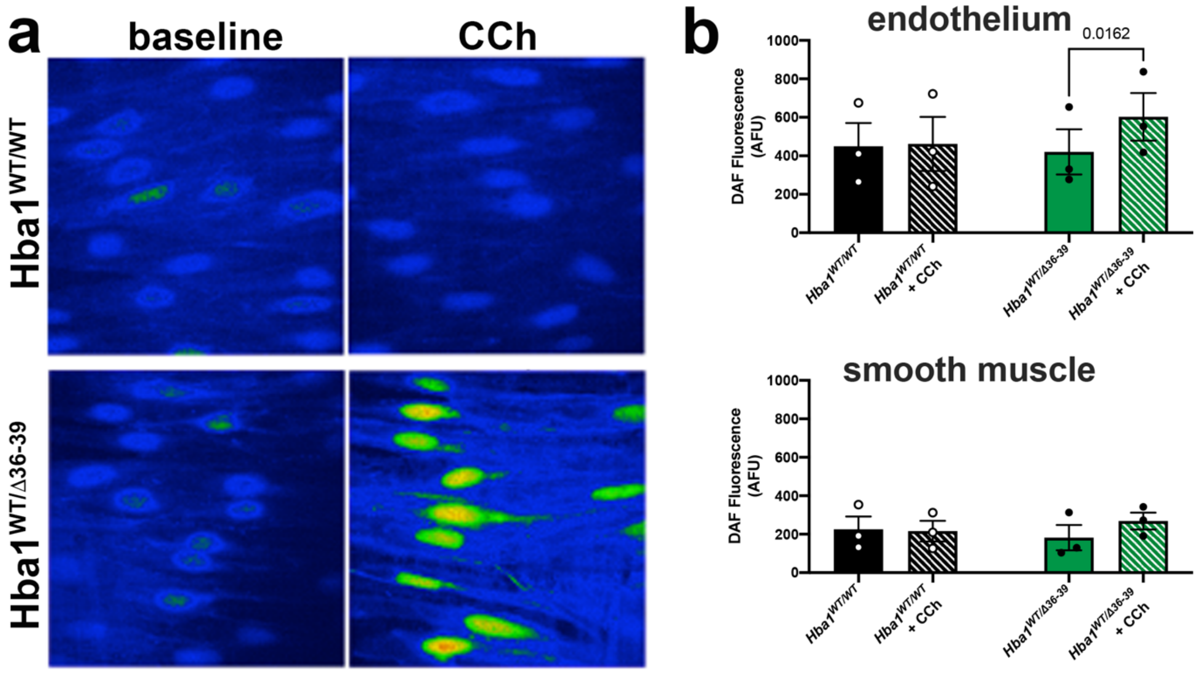

SUPPLEMENTAL FIGURE 11: Increased nitric oxide generation in mice lacking an eNOS binding site in their alpha globin. (**a**) *En face* imaging of third-order mesenteric arterioles that were loaded with DAF and were subjected to 10 μM carbachol (CCh) stimulation and (**b**) the data quantified in the optical slices of endothelium or smooth muscle. Cross-hatched bar graphs represent the CCh-stimulated condition. All measurements were performed with n = 3 mice, with several fields of view in each artery averaged for one replicate value. Littermate groups were compared with a paired t-test. P-values less than 0.05 are shown. Bar graphs are centered on mean with error bars denoting standard error. Source data are provided in the source data file.

Supplemental Figure 12:

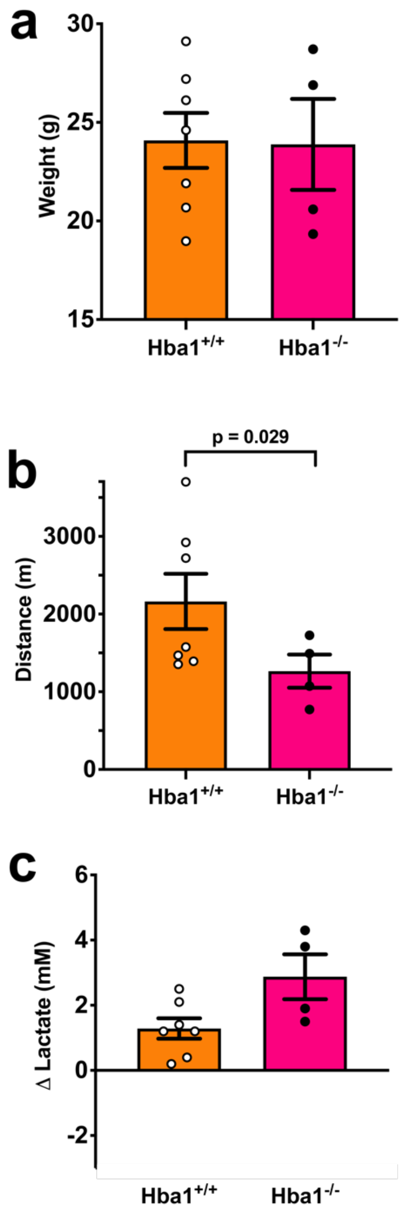

SUPPLEMENTAL FIGURE 12: Global deletion of Hba1 *reduces exercise capacity***.** Mice with a constitutive, global knockout of *Hba1* had similar weight to *Hba1*$^{+/+}$ controls (**a**) but decreased exercise capacity (**b**), with increased blood lactate (**c**). The significant defect in running distance could be related to the anemia observed in these mice (Supplementary Figure 7). For these experiments, n = 7 *Hba1*$^{+/+}$mice and n = 4 and for *Hba1*$^{–/–}$ mice were used. Results were compared

with a one-sided t-test. Bar graphs are centered on mean with error bars denoting standard error.

Source data are provided in the source data file.

Supplemental Figure 13:

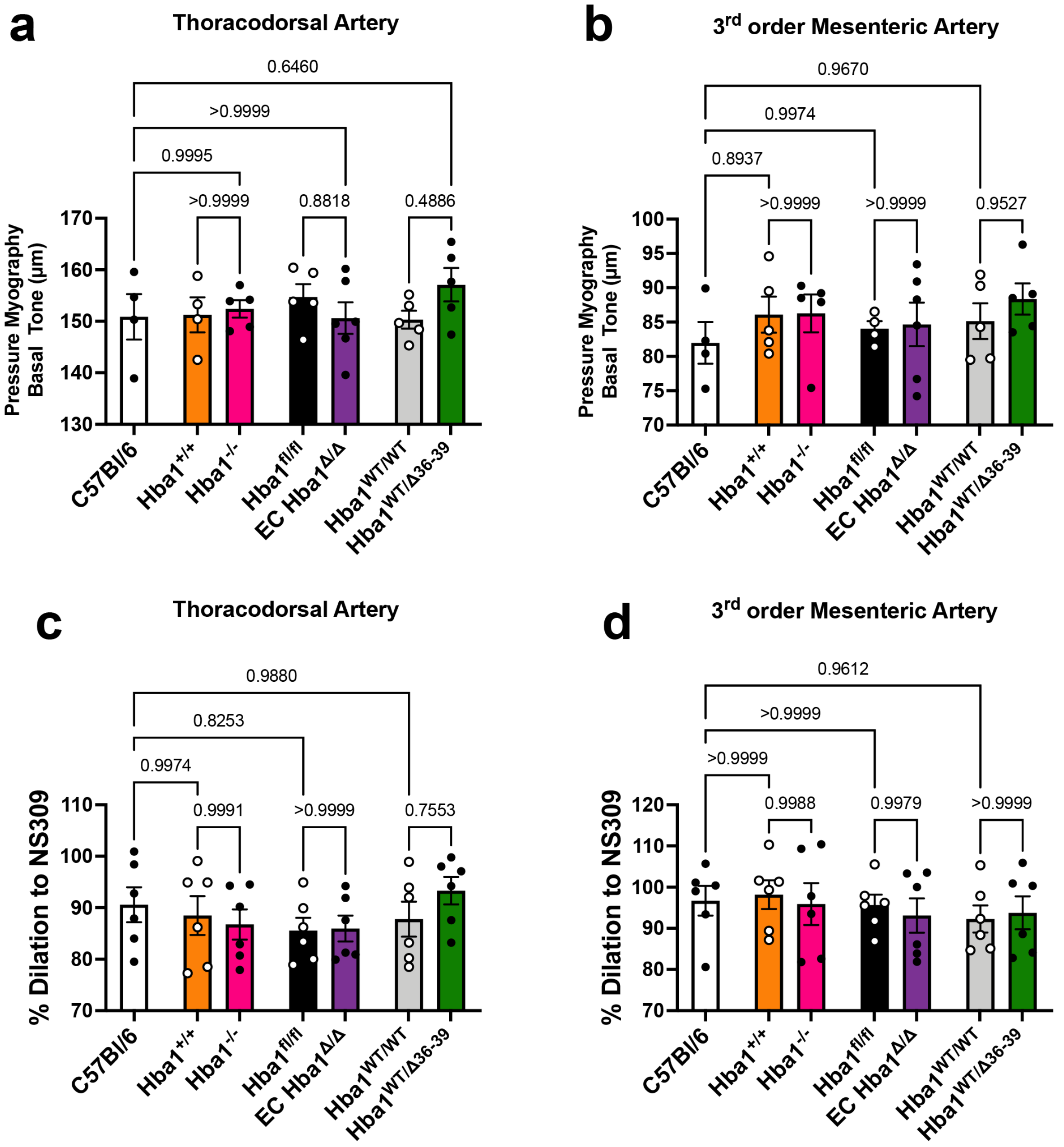

SUPPLEMENTAL FIGURE 13: Basal tone of the vessels was not different for littermate control groups. Basal lumen diameter of the thoracodorsal artery (**a**) and third-order mesenteric artery (**b**) from the experimental genotypes and their littermate controls. (**c, d**) The dilation of thoracodorsal arteries (**c**) and third-order mesenteric arteries (**d**) from the indicated genotypes were subjected to a single dose of 1 µM NS309 to evaluate maximum dilation. The P-values for

comparisons are shown. The groups were compared with a one-way ANOVA. Bar graphs are centered on mean with error bars denoting standard error. Source data are provided in the source data file.

Supplemental Table 1: Genotypes of P21 pups from five breeding pairs of *Hba1*$^{fl/+}$ mice. P-values were determined with a χ^2 test.

***Hba1*$^{fl/+}$ × *Hba1*$^{fl/+}$**				
**Mating Pair**	**Litter**	***Hba1*$^{fl/fl}$**	***Hba1*$^{fl/+}$**	***Hba1*^{WT}**
**1**	1	1	4	2
	2	2	4	2
**2**	1	3	2	3
	2	3	3	2
**3**	1	2	4	2
	2	2	3	2
**4**	1	1	4	1
	2	3	1	3
**5**	1	1	4	2
	2	2	5	1
**Total**		20	34	20
**Percentage**		27	46	27
P-value range: 0.10 - 0.90				

Supplemental Table 2: Genotypes of P21 pups from four breeding pairs of *Hba1*$^{fl/+}$*; Cdh5-Cre*^{ERT2} mice. P-values were determined with a χ^2 test.

***Hba1*$^{fl/+}$*; Cdh5-Cre*^{ERT2} × *Hba1*$^{fl/+}$*; Cdh5-Cre*^{ERT2}**				
**Mating Pair**	**Litter**	***Hba1*$^{fl/fl}$*; Cdh5-Cre*^{ERT2}**	***Hba1*$^{fl/+}$*; Cdh5-Cre*^{ERT2}**	***Hba1*$^{+/+}$*; Cdh5-Cre*^{ERT2}**
**1**	1	1	5	2
	2	3	2	2
**2**	1	2	2	3
	2	3	3	1
	3	3	4	0
**3**	1	1	4	1
	2	1	4	3
	3	4	2	3
**4**	1	0	5	2
	2	1	3	2
**Total**		19	34	19
**Percentage**		26.4	47.2	26.4
P-value range: 0.975 - 0.99				

Supplemental Table 3: Genotypes of P21 pups from three breeding pairs of *Hba1$^{fl/+}$; Sox2-Cre* mice. P-values were determined with a χ^2 test.

Hba1$^{fl/+}$; Sox2-Cre* × *Hba1$^{fl/+}$; Sox2-Cre				
**Mating Pair**	**Litter**	***Hba1$^{fl/fl}$; Sox2-Cre***	***Hba1$^{fl/+}$; Sox2-Cre***	***Hba1$^{+/+}$; Sox2-Cre***
**1**	1	1	5	0
	2	1	3	1
	3	1	3	1
	4	1	3	1
	5	0	3	3
**2**	1	0	3	3
	2	1	3	2
**3**	1	1	3	2
	2	1	3	2
	3	1	3	3
**Total**		8	32	18
**Percentage**		13.7	55.2	31.1
P-value range: 0.1 - 0.9				

Supplemental Table 4: Genotypes of P21 pups from three breeding pairs of *Hba1$^{+/neo}$* mice. P-values were determined with a χ^2 test.

Hba1$^{+/neo}$* × *Hba1$^{+/neo}$				
**Mating Pair**	**Litter**	**Hba1$^{neo/neo}$**	**Hba1$^{+/neo}$**	**Hba1$^{+/+}$**
**1**	1	0	4	2
	2	1	3	1
	3	1	3	2
	4	1	6	0
**2**	1	1	4	2
	2	0	5	3
	3	1	3	1
**3**	1	0	3	3
	2	1	2	2
	3	1	2	3
**Total**		7	35	19
**Percentage**		11.5	57.4	31.1

P-value range: 0.025 - 0.05			

Supplemental Table 5: Genotypes of P21 pups from three breeding pairs of *Hba1*$^{WT/Δ36-39}$ mice. P-values were determined with a χ^2 test.

Hba1$^{WT/Δ36-39}$ × ***Hba1***$^{WT/Δ36-39}$				
**Mating Pair**	**Litter**	***Hba1*** $^{Δ36-39/Δ36-39}$	***Hba1*** $^{WT/Δ36-39}$	***Hba1*** $^{WT/WT}$
**1**	1	0	4	4
	2	0	2	3
	3	0	2	3
	4	0	2	5
	5	0	3	3
**2**	1	0	4	4
	2	0	2	2
	3	0	2	3
	4	0	3	3
**3**	1	1	4	1
**Total**		1	28	31
**Percentage**		1.67	46.67	51.67
P-value < 0.005				
